# Identification of significant biomarkers for predicting the risk of bipolar disorder with arteriosclerosis based on integrative bioinformatics and machine learning

**DOI:** 10.3389/fpsyt.2024.1392437

**Published:** 2024-09-03

**Authors:** Xiabing Zheng, Xiaozhe Zhang, Yaqi Zhang, Cai Chen, Erni Ji

**Affiliations:** ^1^ Department of Bipolar Disorder, Shenzhen Kangning Hospital, Shenzhen Mental Health Center, Shenzhen, Guangdong, China; ^2^ Department of Cardiology, The Eighth Affiliated Hospital of Sun Yat-Sen University, Shenzhen, Guangzhou, China; ^3^ Department of Geriatrics, Shenzhen Kangning Hospital, Shenzhen Mental Health Center, Shenzhen, Guangdong, China; ^4^ Department of Drug Dependence, Shenzhen Kangning Hospital, Shenzhen Mental Health Center, Shenzhen, Guangdong, China

**Keywords:** bipolar disorder, arteriosclerosis, bioinformatics, hub genes, CX3CR1, ST6GAL1

## Abstract

**Introduction:**

Increasing evidence has indicated a connection between bipolar disorder (BD) and arteriosclerosis (AS), yet the specific molecular mechanisms remain unclear. This study aims to investigate the hub genes and molecular pathways for BD with AS.

**Methods:**

BD-related dataset GSE12649 were downloaded from the Gene Expression Omnibus database and differentially expressed genes (DEGs) and key module genes derived from Limma and weighted gene co-expression network analyses (WGCNA) were identified. AS-related genes were sourced from the DisGeNET database, and the overlapping genes between DEGs and AS-related genes were characterized as differentially expressed arteriosclerosis-related genes (DE-ASRGs). The functional enrichment analysis, protein-protein interaction (PPI) network and three machine learning algorithms were performed to explore the hub genes, which were validated with two external validation sets. Additionally, immune infiltration was performed in BD.

**Results:**

Overall, 67 DE-ASRGs were found to be overlapping between the DEGs and AS-related genes. Functional enrichment analysis highlighted the cancer pathways between BD and AS. We identified seven candidate hub genes (CTSD, IRF3, NPEPPS, ST6GAL1, HIF1A, SOX9 and CX3CR1). Eventually, two hub genes (CX3CR1 and ST6GAL1) were identified as BD and AS co-biomarkers by using machine learning algorithms. Immune infiltration had revealed the disorder of immunocytes.

**Discussion:**

This study identified the hub genes CX3CR1 and ST6GAL1 in BD and AS, providing new insights for further research on the bioinformatic mechanisms of BD with AS and contributing to the diagnosis and prevention of AS in psychiatric clinical practice.

## 1 Introduction

Bipolar disorder (BD) is marked by alternating episodes of depression as well as either mania or hypomania, impacting an estimated 40 million individuals worldwide (1). Bipolar disorder is the 17th leading cause of global burden of diseases (2) and often lead to functional impairment and reduced quality of life (3). Psychiatric and nonpsychiatric medical comorbidities are common in patients with BD and might also contribute to increased mortality, particularly from cardiovascular diseases (CVDs) (4). Individuals diagnosed with bipolar disorder exhibit a notably elevated risk of mortality from CVDs compared to the general population, with a standardized mortality ratio of 1.73 and experience cardiovascular mortality occurring an average of 17 years earlier (1, 5, 6). This elevated risk persists even after considering the high prevalence of cardiovascular risk factors present in individuals with bipolar disorder (7). Evidence from a large epidemiological study suggests that the elevated occurrence and premature onset of CVDs in BD surpasses what can be accounted for by traditional cardiovascular risk factors (8).

Arteriosclerosis (AS) is primarily an aging-related process characterized by increased stiffness in elastic arteries, including the aorta (9). The pathological characteristics of AS include elastin fracture, an increase in collagen fibers, and calcium deposition. AS develops as a result of increased production or progressively greater engagement of stiffer load-bearing elements in the arterial wall, such as collagen (10). Arterial stiffness of AS is a significant risk factor for CVDs and a powerful predictor of CVDs morbidity and mortality (11).

Several studies have investigated the phenomenon between AS and BD. In a study investigating the relationship between arterial stiffness and BD, patients were found to have higher arterial stiffness compared to healthy controls regarding to the elastic modulus of the carotid artery (12). Furthermore, research suggested that patients with BD have greater carotid intima-media thickness before middle age and were at increasing risk of atherosclerosis (13). BD was closely associated with and independently contribute to increasing atherogenic potential (14). Long-term depressive and manic symptom burden, especially the persistence and duration of mood syndromes, has been independently linked to poor endothelial function and impaired vascular function, which lead to subsequent cardiovascular morbidity and mortality (15–17). The chronicity of mood symptoms contributes to vasculopathy in a dose-dependent fashion, and patients with more manic/hypomanic symptoms had poorer endothelial function (17, 18). Besides, lipid abnormalities that contribute to an increased atherogenic potential are implicated in the pathophysiology of BD (19). Underlying pathophysiological factors such as immune-inflammatory abnormalities, hypothalamic-pituitary-adrenal axis and sympathomedullary hyperactivity, increased platelet reactivity, reduced heart rate variability, oxidative stress, and endothelial dysfunction may also contribute to the heightened risk of CVDs (20–22).

Most studies focusing on the relationship between BD and AS are based on clinical investigation and examination, and scarce data are available to explore associated pathological mechanisms and genetic alterations. With the advancement of multi-omics technologies, researchers are now able to uncover new insights into human diseases by identifying biomarkers, pathways, and other approaches, thereby offering novel strategies for disease diagnosis and treatment (23). Genome-wide association studies (GWAS) have identified several genetic variants between BD and CVDs (24, 25). The risk of myocardial infarction has been linked to a genetic variant in the ITIH3–ITIH4 genes, which have also been implicated in the risk of BD (Consortium, 2011). A phenome-wide association study (PheWAS) exploring BD and susceptibility genetic variants across various medical conditions indicated that the genome-wide single nucleotide polymorphism (SNP) rs4765913 in the CACNA1C gene may be linked to an increased risk of “cardiovascular dysgenesis” (25). An existing study of genome wide and candidate gene studies related to cardiometabolic diseases and mood disorders revealed 24 potential pleiotropic genes that are likely to be shared between mood disorders and cardiometabolic diseases risk (26). Moreover, data mining and machine learning techniques have been employed in the study of complex diseases to identify potential biomarkers (27). However, there are limited biologically relevant diagnostic markers available on AS in BD. Exploring new markers and methods to assess AS risk in BD patients could lead to more accurate diagnosis and improved treatment of BD. Investigating the pathogenesis and associations with AS is of considerable interest in the field of psychiatry and this progress requires having better understanding of molecular psychopathology and specific biomarkers (28). Therefore, the present study was conducted to predict the bidirectional hub genes and related pathways between BD and AS using bioinformatics and machine learning algorithms.

## 2 Methods

### 2.1 Data collection and preparation

Gene datasets related to bipolar disorder were downloaded from the Gene Expression Omnibus (GEO) database (https://www.ncbi.nlm.nih.gov/geoprofiles/). The inclusion criteria are set as: expression profiles come from the same sample source and should contain enough sample size that at least fifty samples to ensure accuracy. In addition, the test specimens included should be from humans. Finally, two microarray datasets [GSE12649 and GSE5392] derived from prefrontal cortex were downloaded from GEO (Affymetrix GPL96 platform, Affymetrix Human Genome U133A Array). The GSE12649 comprised 33 bipolar disorder samples and 34 control groups, while the GSE5392 contained 30 bipolar disorder samples and 52 control groups. GSE12649 was selected as the training group and GSE5392 as the test group. The expression matrix file of GSE12649 was normalized through the Limma package (29).

**Figure 1:**
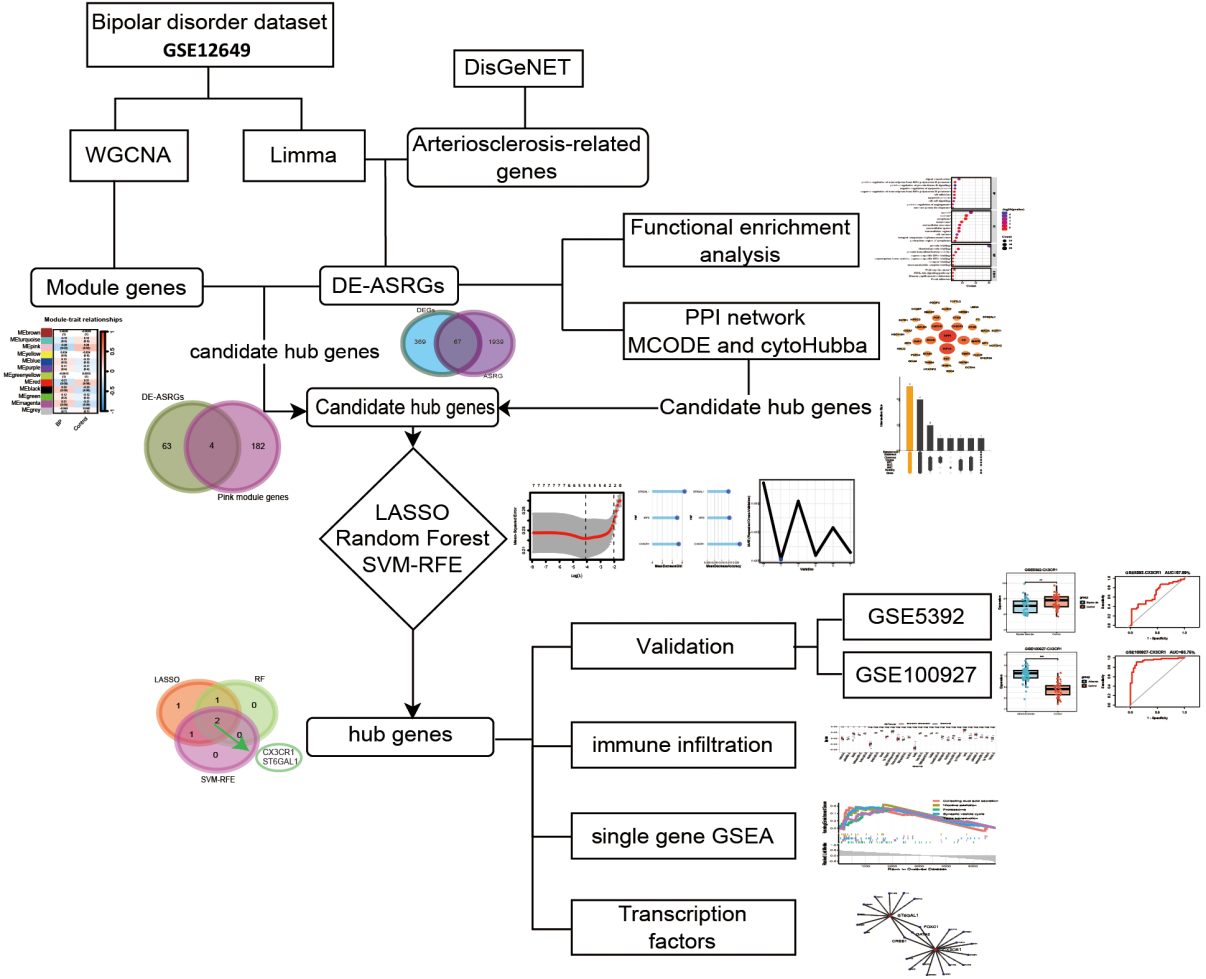
The flow diagram for the whole study.

Since no microarray data sets detecting gene expression in brain especially in prefrontal cortex among arteriosclerosis, DisGeNET (https://www.disgenet.org/home/) was used to retrieve 2006 arteriosclerosis-related genes (ASRGs) (**Supplementary Table S1**). The DisGeNET database is a comprehensive gene-associated information platform that integrates experimentally validated data with information from authoritative repositories and scientific literature to present genes and variants linked to human diseases (30). Given that the GEO database lacks datasets directly related to AS, and considering that atherosclerosis is a consequence of arteriosclerosis (10), we have chosen the atherosclerosis dataset GSE100927 as our test group. The whole process flow is shown in **Figure 1** and the complete information dataset is given in **Table 1**.

**Table 1:** Detailed data set information.

	Dataset	Platform	Disease	Samples (patients/controls)	Source
Discovery cohort	GSE12649	GPL96	Bipolar disorder	33/34	Prefrontal cortex
Validation cohort	GSE5392	GPL96	Bipolar disorder	30/52	Prefrontal cortex
Validation cohort	GSE100927	GPL17077	Atherosclerosis	69/35	Artery

### 2.2 Differential gene expression analysis

After preparing the data, differential expression analysis was performed using the limma package in R (version 4.3.1) on the GSE12649 dataset. Differentially expressed genes (DEGs) were identified which were differentially expressed between the BD and control groups. The DEG threshold was set as P value <0.05 and |log2FC (fold change) |>0.2. Next, the difference analysis results were presented using the heatmap and volcano plot. In both plots, blue indicated low expression, and red indicated high. Then, the online Venn diagram tool (http://bioinformatics.psb.ugent.be/webtools/Venn/) was used to obtain the differentially expressed arteriosclerosis-related genes (DE-ASRGs) between DEGs and ASRG.

### 2.3 Functional enrichment analysis of DE-ASRGs

To further understand the function of the DE-ASRGs, we performed Gene Ontology (GO) enrichment analysis (31) and Kyoto Encyclopedia of Genes and Genomes (KEGG) pathway enrichment analysis (32) using the DAVID (https://david.ncifcrf.gov/). GO is a structured way to represent biological functions in terms of core entities and annotate protein biomarkers in the biological process (BP), cellular composition (CC) and molecular function (MF) levels. KEGG enables the correlation of gene catalogues to system functions at the cellular, species and ecosystem levels, facilitating researchers to understand the signaling pathways in which genes are involved. Based on the analysis, we selected the enrichment analysis results with a count greater than 5 and arranged them in descending order of count to identify the top 10.

### 2.4 Construction of protein–protein interaction network and identification of candidate hub genes

The DE-ASRGs were analyzed by a search tool for the retrieval of interacting genes/proteins (STRING) (https://www.string-db.org/) to predict the PPI network and to determine the possible relationships between them (confidence level 0.4). The STRING database and Cytoscape software (v3.10.1) completed constructing the PPI network for DE-ASRGs. Gene modules were analyzed and network characteristics of genes were ranked by protein score via the MCODE and CytoHubba plugin, respectively. The CytoHubba plugin was used to score each node gene by 9 randomly selected algorithms, including MNC (Maximum Neighbourhood Component), Degree, MCC (Maximal Clique Centrality), EPC (Edge Percolated Component), Closeness, BottleNeck, Betweenness, Radiality, and Stress. The top 10 genes from each algorithm were used to screen candidate hub genes through the “UpSetR” package.

### 2.5 Screening of target genes by weighted gene co-expression network analysis

The WGCNA was utilized for constructing unsigned co-expression networks for the identification of co-expression modules across samples using the “WGCNA” R package (33). WGCNA is a highly efficient and accurate method for analyzing microarray data, and using this approach can help identify genes associated with diseases. Initially, normalized mRNA expression data were used to perform WGCNA to identify gene co-expression and the correlation between gene modules and clinical characteristics (BD compared to control groups). Samples were examined for missing values and then clustered using the average linkage hierarchical method. Afterward, the optimal values of the weighted parameters of the adjacent functions were obtained using the pickSoftThreshold function and were used as soft thresholds for subsequent network construction. Moreover, a topological overlap matrix (TOM) was devised on the basis of an adjacency matrix, and a dynamic tree-cutting algorithm was used for detecting gene modules with a minimum gene group size of 30 and power = 6. The correlation of each module with the BD was calculated, and the module with P<0.05 was defined as the key module. Finally, we took the intersection between DE-ASRGs and WGCNA-derived key module genes to obtain potential hub gene of AS and BD.

### 2.6 Machine learning algorithms

To further identify hub genes from the potential candidates, machine learning algorithms were employed. To ensure the repeatability of these algorithms, we set the seed at 2023. The Least Absolute Shrinkage and Selection Operator (LASSO) algorithm was executed using the glmnet package (34), with a tenfold cross-validation was performed to adjust the optimal penalty parameter. The response type was set to binomial, nlambda was set to 100, and alpha was fixed at 1. Moreover, we chose the best lambda value by “lambda.min”. Subsequently, the Random Forest (RF) algorithm (35) was implemented using the randomForest package. We explored the optimal number of random forest trees using cross-validation errors and settled on 500 trees for analysis. The significance of genes in the RF model was evaluated based on mean decrease accuracy and mean decrease Gini. The intersection genes from the top 3 mean decrease accuracy and top 3 mean decrease Gini were identified. Additionally, the support vector machine recursive feature elimination (SVM-RFE) method (36), acting as a vigilant machine learning approach, was employed to select the most relevant variables by eliminating SVM-produced eigenvectors. For the categorization analyses of the screened markers in the BD, the caret package was utilized to conduct the SVM-RFE. The results of SVM-RFE were visualized, and through ten-fold cross-validation, the blue point represented the maximum classification accuracy, highlighting the corresponding gene sets as the most effective diagnostic markers. Only the names of valuable genes used in each machine learning model were included, irrespective of the specific learning model. The hub genes were determined as the intersection genes of valuable genes obtained from the three machine learning algorithms.

SVMs have been shown to exhibit robust classification performance and high accuracy when utilizing feature selection genes in the analysis of microarray expression profiles (37). In this study, SVM was used to evaluate the predictive capability of hub genes as feature genes as follows: 1) the BD datasets including GSE12649 and GSE5392 were separately divided into training (70%) and test (30%) sets; 2) SVM modeling was conducted using gene expression data of hub genes from the training set; 3) optimal SVM hyperparameters were determined through ten-fold cross-validation; 4) model accuracy was assessed using the test set.

### 2.7 Verification of hub genes

To validate the expression of two hub genes in BD and AS, the t-test was employed to analyze their expression levels. Initially, the expression levels of these hub genes were measured in the dataset GSE12649. Subsequently, two hub genes were validated in the independent datasets GSE5392 and GSE100927. The expression levels of the hub genes were visually represented in boxplots generated using the “ggplot2” package in R. Furthermore, the predictive and discriminatory abilities of the hub genes were evaluated through receiver operating characteristic (ROC) analysis using the “pROC” package, with the area under the curve (AUC) values determined.

### 2.8 Single gene GSEA analysis

Following the identification of hub genes, single-gene gene set enrichment analysis (GSEA) was conducted to uncover their potential functions, utilizing the “clusterProfiler” package. This analysis was carried out using the human reference genome. Subsequently, enrichplot was utilized to visualize the top 5 activating and inhibiting pathways for each gene in the two disease groups.

### 2.9 Correlation analysis between infiltrating immune cells and hub genes

For immune infiltration analysis, single-sample gene set enrichment analysis (ssGSEA) was carried out using the “GSVA” package to evaluate the relative infiltration levels of 28 immune cell types in each sample of BD. A comparison of immune cell content between the BD and control groups was conducted using the Wilcoxon test. Furthermore, Spearman correlation analysis between infiltrating immune cells and hub genes was performed using the “corrplot” package in R. The resulting correlations between hub genes and immune cells were visualized using lollipop plots.

### 2.10 Statistical analysis

R software version 4.2.1 was used to perform statistical analyses. Using the Student’s t-test, continuous variables were compared between two groups. A p-value less than 0.05 was statistically significant.

## 3 Results

### 3.1 Screening of differentially expressed genes in BD and identification of arteriosclerosis-related DEGs

**Figure 2:**
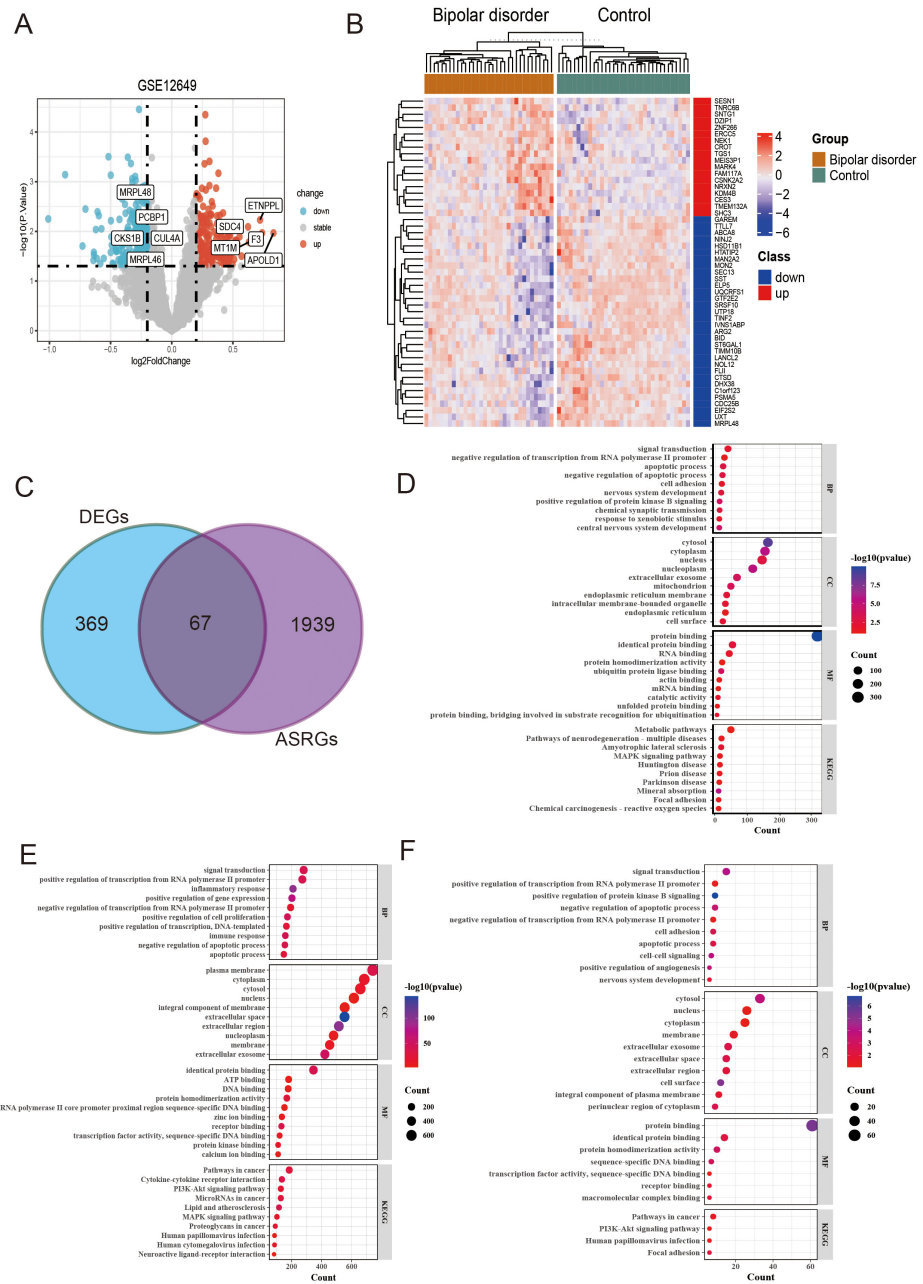
Identification and analysis of differentially expressed genes (DEGs) and differentially expressed arteriosclerosis-related genes (DE-ASRGs) in bipolar disorder (BD). **(A)** Volcanic map of the DEGs. **(B)** The expression patterns of top 50 DEGs shown by heatmap. **(C)** Venn diagram of DE-ASRGs. **(D-F)** The bubble plot of GO enrichment and KEGG pathway analysis results for DEGs **(D)**, ASRG **(E)**, and DE-ASRGs **(F)**.

After standardizing the microarray results, the differentially expressed genes (DEGs) were screened by “Limma” package (p < 0.05 and |log FC| > 0.2). The GSE12649 dataset contained 436 DEGs, including 201 upregulated genes and 235 downregulated genes (**Figures 2A, B**). Then, we intersected the resulting data with 2006 ASRGs and a total of 67 differentially expressed arteriosclerosis-related genes (DE-ASRGs) were identified (**Figure 2C**).

### 3.2 Analysis of the functional characteristics of DE-ASRGs

In order to analyze the biological functions and pathways, perform GO and KEGG pathway enrichment analysis on the DEGs, ASRG, and DE-ASRG genomes using DAVID (**Figures 2D-F**). Upon comparing the enrichment results of the three genomes, we observed that DE-ASRGs were primarily focused on signal transduction in biological processes. Additionally, negative regulation of apoptotic processes, negative regulation of translation from RNA polymerase II (pol II) promoter, and apoptotic processes were identified as common biological processes across the three genomes. The GO-CC pathways were principally associated with the cytosol, nucleus, and cytoplasm. The GO-MF analysis revealed enrichment in protein binding, identical protein binding and protein homodimerization activity. Additionally, KEGG analysis showed that these DE-ASRGs were enriched in pathways in cancer, PI3K-Akt signaling pathway and human papillomavirus infection (HPV).

### 3.3 Construction of PPI network of DE-ASRGs and potential hub gene screening

**Figure 3:**
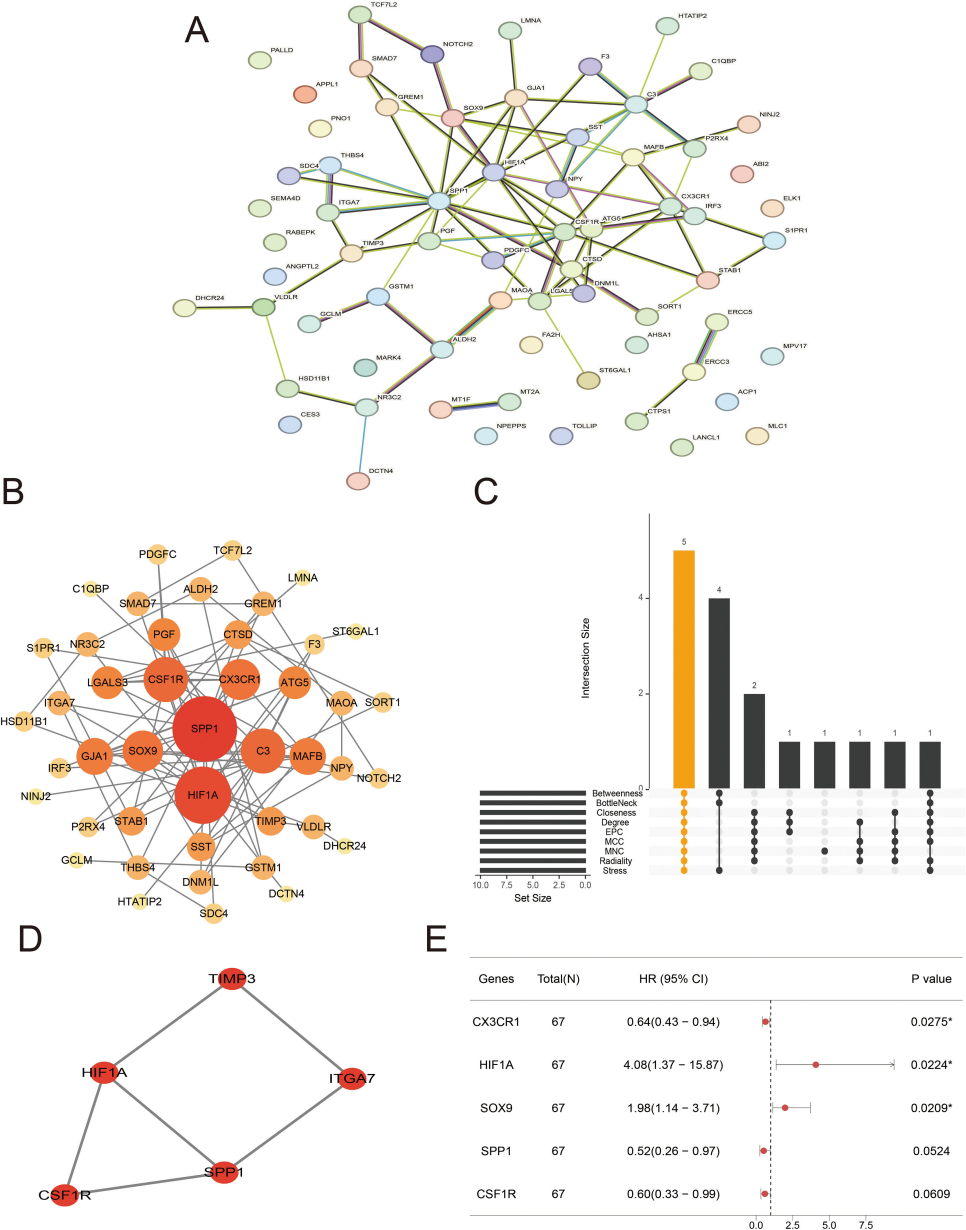
Protein–protein interaction (PPI) network and candidate hub genes. **(A, B)** Interaction network of differentially expressed arteriosclerosis-related genes (DE-ASRGs) by STRING **(A)** and Cytoscape **(B)**. **(C)** The upset plot displaying the common genes among 9 algorithms. **(D)** Cluster identified by MCODE plugin. **(E)** Regression analysis of common genes.

To explore the protein interactions among DE-ASRGs, we performed a PPI network containing 67 nodes and 82 edges with medium confidence (score > 0.4) using the STRING database and visualized the network with Cytoscape software, as shown in **Figures 3A, B**. Subsequently, the MCODE plugin identified a cluster comprising 5 nodes (CSF1R, HIF1A, TIMP3, ITGA7, SPP1) as illustrated in **Figure 3C**. The cytoHubba plugin was used to score each node gene by 9 randomly selected algorithms and the top 10 hub genes from each algorithm were identified. Five common genes (CSF1R, SPP1, HIF1A, SOX9, and CX3CR1) were selected from the 9 algorithms, as shown in **Figure 3D**, using the “UpSetR” package. Further screening of common genes through regression analysis resulted in the identification of three candidate hub genes (HIF1A, SOX9 and CX3CR1) as depicted in **Figure 3E**.

### 3.4 Construction of co-expression networks

**Figure 4:**
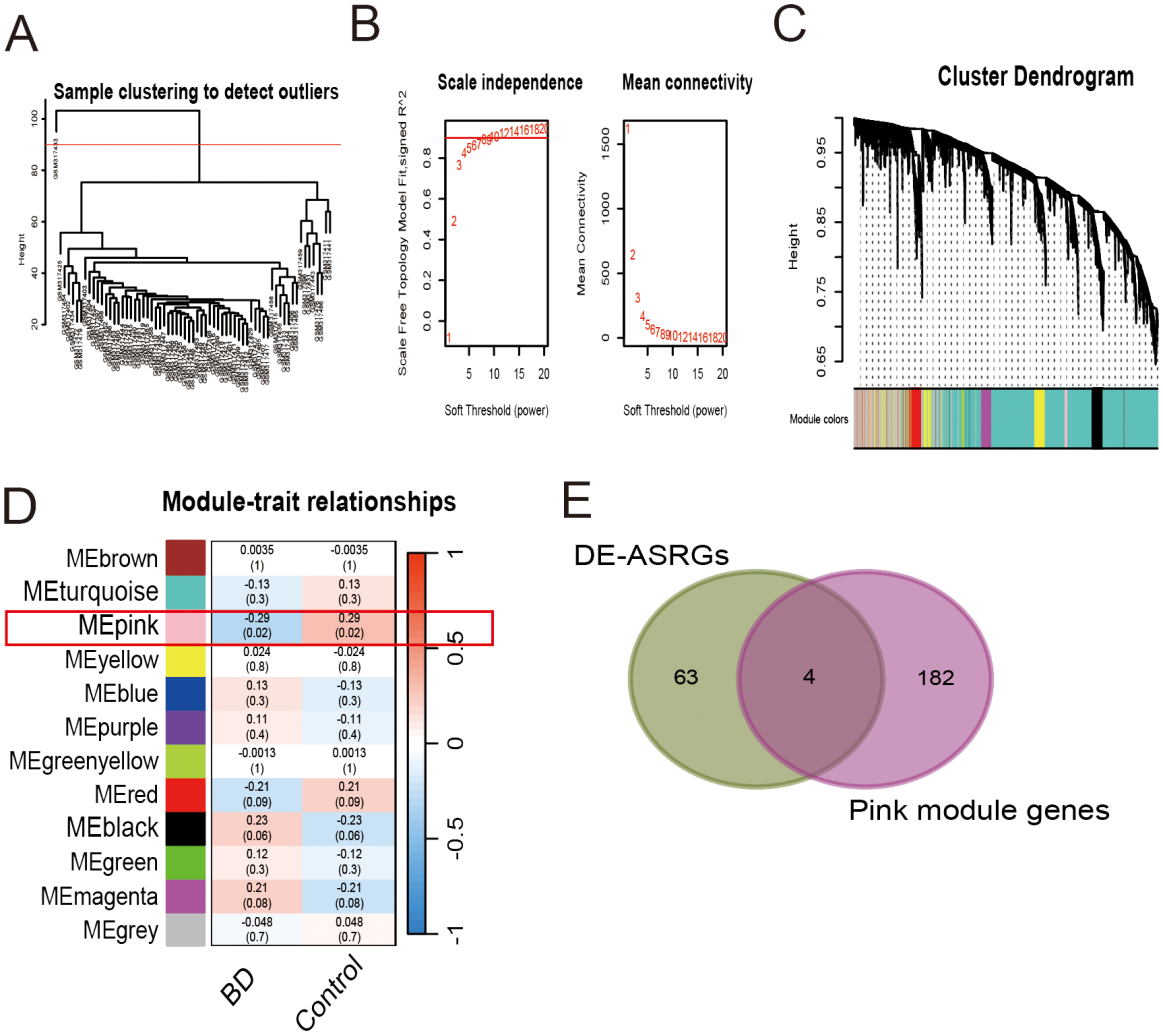
Weighted gene co-expression network analysis for gene set GSE12649. **(A)** Sample clustering to detect outliers. **(B)** Scale independence and mean connectivity. **(C)** Cluster dendrogram. **(D)** Module-trait relationships. **(E)** Venn diagram of 4 candidate hub genes.

In this study, we conducted WGCNA to identify gene modules associated with BD in the GSE12649 dataset. Initially, the dataset underwent outlier screening, followed by clustering of the remaining samples as depicted in **Figure 4A**. To establish scale-free networks, a soft-threshold power of β = 6 (yielding a scale-free R^2^ of 0.85) was selected for BD, as shown in **Figure 4B**. Using a minimum module size of 30, we identified 12 modules, each represented by a unique color (**Figure 4C**). Heat maps illustrating module-trait relationships based on Pearson correlation coefficients were generated to evaluate the connection between each module and BD (**Figure 4D**). Among the 11 modules analyzed, the pink module exhibited the strongest correlation with BD (correlation coefficient = -0.29, P = 0.02), encompassing a total of 186 genes. Subsequently, the 186 genes within the pink module were intersected with the DE-ASRGs, resulting in the identification of 4 candidate hub genes (CTSD, IRF3, NPEPPS and ST6GAL1) (**Figure 4E**). These candidate hub genes were then integrated with those identified through Cytoscape analyses, ultimately yielding a total of 7 candidate hub genes (CTSD, IRF3, NPEPPS, ST6GAL1, HIF1A, SOX9 and CX3CR1).

### 3.5 Screening hub genes through machine learning

**Figure 5:**
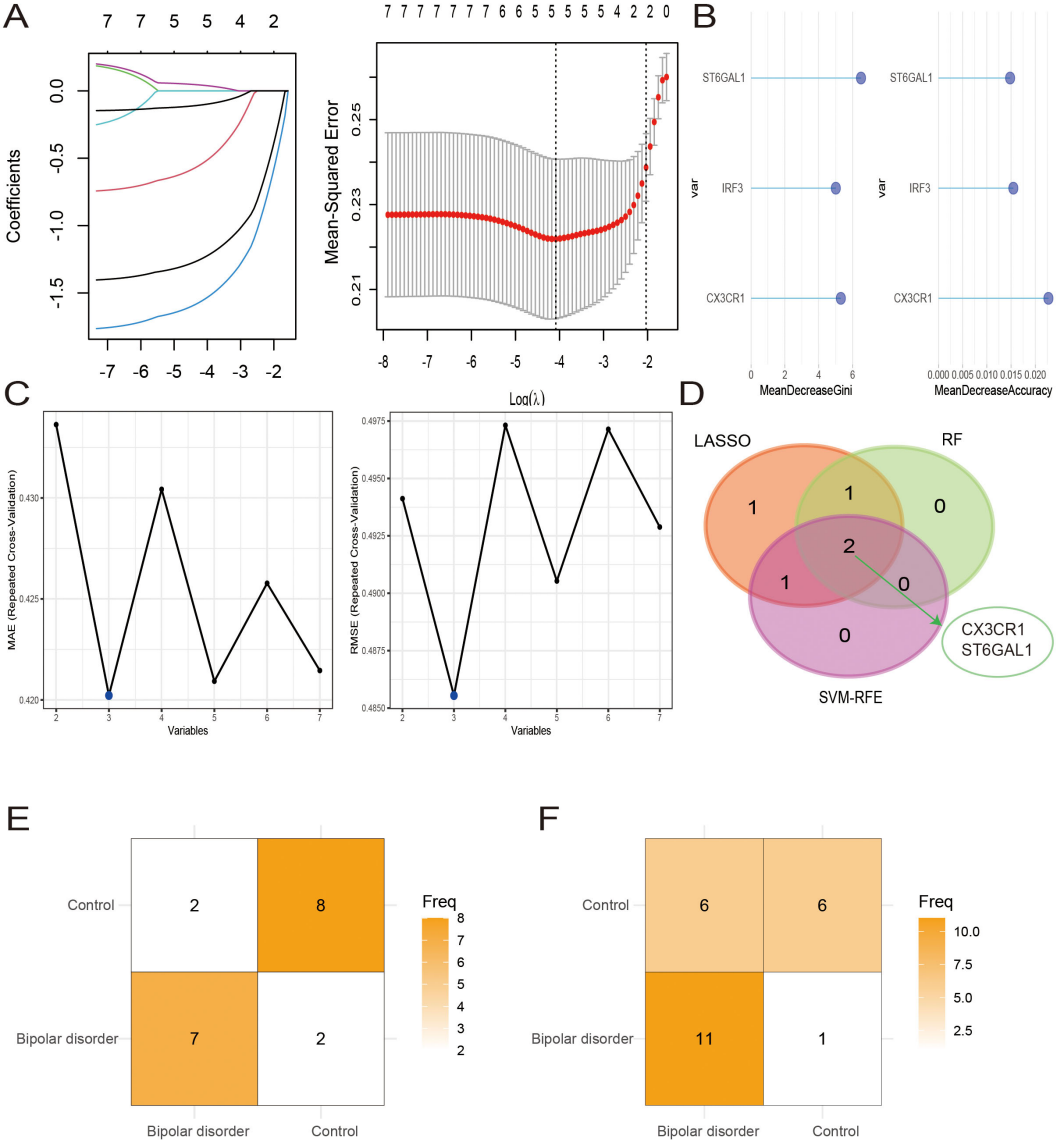
Hub cross-talk gene screening. **(A-C)** Results of Least Absolute Shrinkage and Selection Operator (LASSO) **(A)**, Random Forest (RF) **(B)**, and support vector machine recursive feature elimination (SVM-RFE) **(C)** analysis. **(D)** Venn diagram of 2 hub genes. **(E, F)** The confusion matrices for the model’s validation on the GSE12649 **(E)** and GSE5392 **(F)** datasets.

Lasso regression method, random forest and SVM-RFE method were used to further screen the expression matrix of the 7 candidate hub genes. The LASSO regression model was designed based on BD as well as control samples. By analyzing the Lasso coefficient profiles and selecting the optimal tuning parameter, λ was determined to be 0.0168688 (**Figure 5A**). Subsequently, five candidate genes were identified through the Lasso analysis. Following this, the seven candidate hub genes were fed into the RF classifier, and the top three genes were chosen based on the mean decrease accuracy and mean decrease Gini scale. Three genes were selected as candidate genes from the RF results (**Figure 5B**). Furthermore, SVM-RFE analysis was performed, revealing that the model incorporating three genes exhibited the best Mean Absolute Error (MAE) and Root Mean Square Error (RMSE) (**Figure 5C**). By comparing the overlapping genes obtained from three machine learning methods, two hub genes were selected: CX3CR1 and ST6GAL1 (**Figure 5D**).

We developed SVM models using the BD datasets GSE12649 and GSE5392 to assess the predictive capability of the two hub genes, CX3CR1 and ST6GAL1. The model’s performance on the GSE12649 test set showed an accuracy of 78.90% and a Kappa of 0.58. For the GSE5392 test set, the SVM model achieved an accuracy of 70.83% and a Kappa of 0.42. The SVM results are detailed in **Figures 5E, F**, as well as in **Table 2**.

**Table 2:** Classification performance of the two hub genes in the GSE12649 and GSE5392 dataset.

Dataset	Accuracy	Kappa	P-Value [Acc > NIR]	95% Confidence Interval
GSE12649	78.90%	0.58	0.02	(0.54, 0.94)
GSE5392	70.83%	0.42	0.03	(0.49, 0.87)

### 3.6 The expression analysis and ROC curve analysis of hub genes

**Figure 6:**
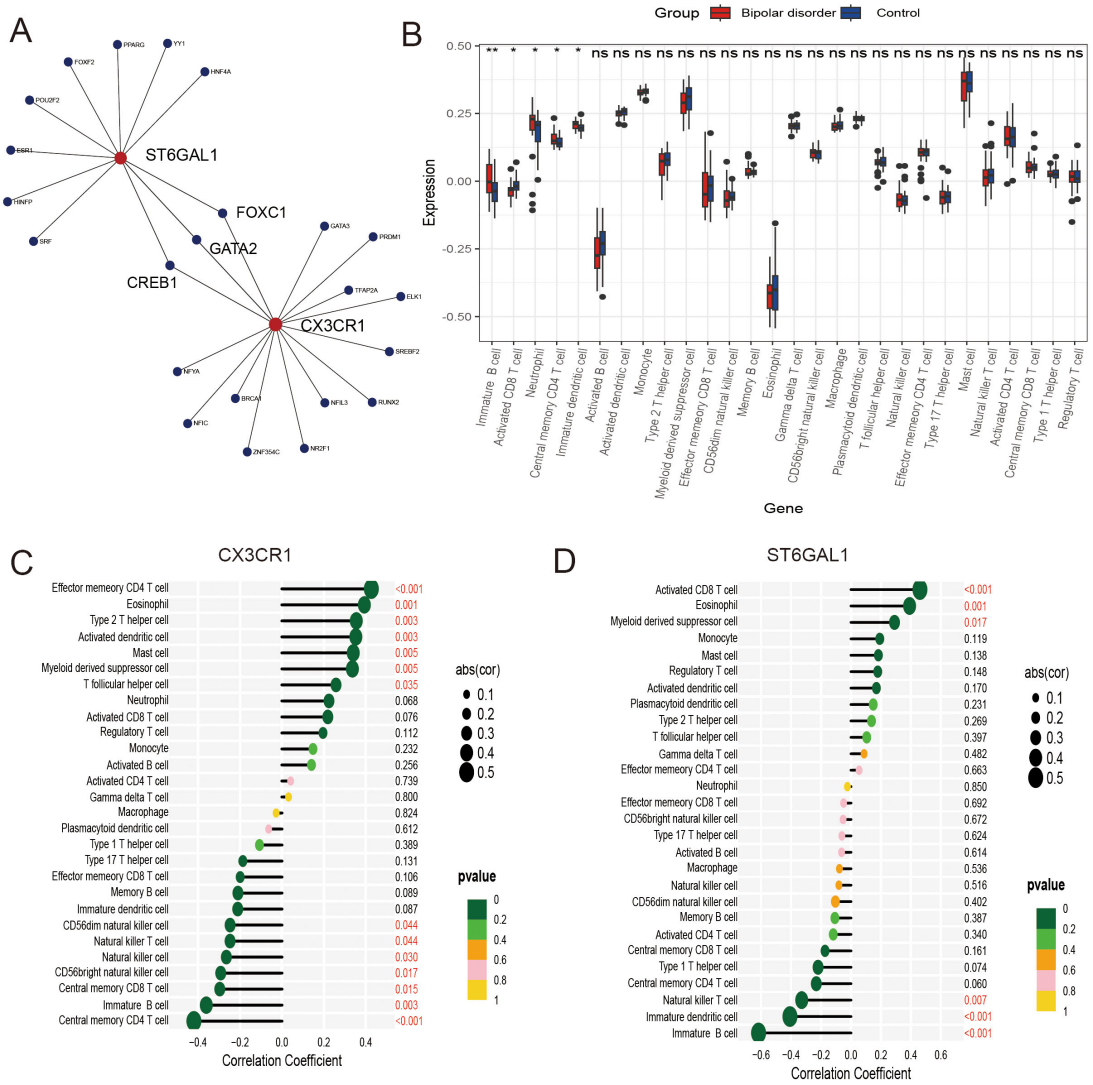
Regulatory networks and immune infiltration analysis for hub genes. **(A)** Transcription factor-key gene interaction network. **(B)** The difference in immune infiltration between bipolar disorder samples and control samples. **(C, D)** Correlation between CX3CR1 and ST6GAL1 with immune infiltrating cells.

**Figure 7:**
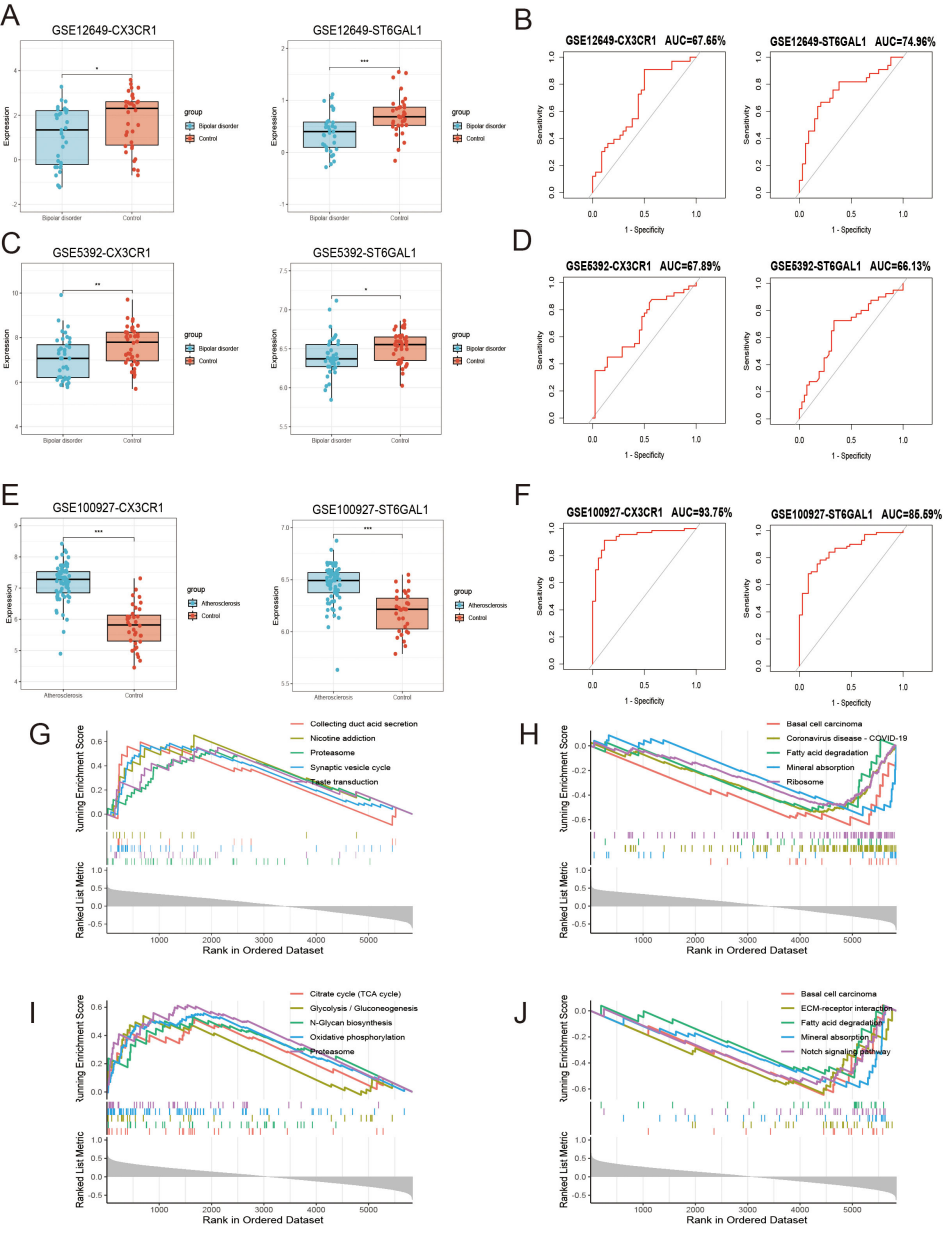
Hub gene expression level, receiver operating characteristic (ROC) analysis and the functions of hub genes including CX3CR1 and ST6GAL1. **(A, C, E)** Expression levels of hub genes in GSE12639 **(A)**, GSE5392 **(C)**, GSE100927 **(E)**. **(B, D, F)** The ROC curve analysis of hub genes in GSE12649 **(B)**, GSE5392 **(D)**, GSE100927 **(F)**. **(G-J)** The activating and inhibiting pathways of CX3CR1 **(G, H)** and ST6GAL1 **(I, J)** using GSEA analysis.

We investigated the expression of these two genes in the GSE12649 dataset and GSE5392 validation dataset, comparing BD and control samples. As shown in **Figure 6A**, CX3CR1 and ST6GAL1 showed a significantly lower expression in BD, so as the GSE5392 validation dataset (**Figures 7A, C**). Likewise, we used the AS dataset GSE100927 for validation, and our findings revealed that the expression level of hub genes was higher in the AS group (**Figure 7E**). ROC analysis was used to verify the specificity and sensitivity of the two hub genes for BD and AS diagnosis. The AUC areas of the two hub genes in GSE12649 and GSE5392 were around 0.7, indicating their diagnostic value (**Figures 7B, D**). In GSE100927 dataset, the AUC areas of two hub genes were > 0.8, showing that the hub genes had good diagnostic values.

### 3.7 Single-gene GSEA analysis and identification of transcription factors

Single-gene GSEA analysis was performed on these two hub genes to obtain the related pathways of each gene. The results showed its correlation with proteasome, basal cell carcinoma, fatty acid degradation, mineral absorption and so on (**Figures 7G-J**). The interaction network consisted of two hub genes and 23 TFs (Transcription Factors) (**Figure 6A**). ST6GAL1 was regulated by eleven TFs, while CX3CR1 was regulated by fifteen TFs. Moreover, FOXC1, GATA2, and CREB1 were found to interact with both hub genes, indicating a potential close interaction between these TFs and the hub genes.

### 3.8 Correlation analysis between hub genes and immune cells

To further understand the involvement of the hub genes in immune infiltration, we conducted Spearman correlation analysis to investigate the potential relationships between these hub genes and immune cell infiltration. The results showed significantly higher levels of immature B cells, neutrophils, central memory CD4 T cells, and immature dendritic cells, as well as significantly lower levels of activated CD8 T cells in the BD group compared to the control group, as seen in the box plot (**Figure 6B**). Our findings regarding the hub genes, CX3CR1 and ST6GAL1, were consistently exhibited significantly positive correlations with eosinophils, while demonstrating significantly negative correlations with immature B cells (**Figures 6C, D**).

## 4 Discussion

BD is a chronic and disabling affective disorder with significant high rate of physical comorbidities. Taking into account efficacious psychopharmacological treatment and adequate follow-up regarding physical comorbidities is critical for reducing the cardiovascular disease risk in patients with BD. BD is known to be a risk factor for accelerated early CVDs (20). Characterized by arterial stiffness, AS may play a role in increasing the risk of CVDs in patients with BD and should be further investigated (12). While the clinical relationship between BD and AS has been well-discussed (12, 18, 38), the pathogenesis underlying this relationship are not fully understood. Therefore, it is essential to explore the molecular changes of these two diseases and provided a theoretical basis for the in-depth understanding of the pathogenesis.

In our study, we identified 67 overlapping DE-ASRGs between BD patients and healthy controls, based on analysis of microarray datasets. Our GO functional enrichment analyses revealed that these DE-ASRGs were closely linked to various biological processes, including signal transduction, positive and negative regulation of translation from RNA pol II promoter, apoptotic processes, cell adhesion, and positive regulation of protein kinase B signaling. These findings indicated that the DE-ASRGs played crucial roles in intracellular signaling and regulatory processes, influencing cell survival, proliferation, and adaptation. Furthermore, our KEGG enrichment analysis showed that the DE-ASRGs were predominantly associated with the cancer pathway, PI3K-Akt signaling pathway, HPV, and focal adhesion. Recent studies have found that the PI3K-Akt pathway plays a crucial role in cerebrovascular diseases, metabolic syndrome, and mental disorders. The PI3K-Akt pathway can serve as a therapeutic target for brain aging and neurodegenerative changes (39). One study suggests that there may be a connection between bipolar disorder and obesity through the PI3K-Akt pathway (40). The PI3K-Akt pathway and focal adhesion, as potential genetic defects in BD and therapeutic targets of lithium, play a role in axonal growth and neuronal development (41). Previous studies have found that lithium is a protective factor for CVDs (42). Based on the founding of this study, the PI3K-Akt pathway and focal adhesion may be potential targets for lithium to reduce the risk of CVDs in BD patients. Interestingly, considering the correlation between PI3K-Akt signaling pathway (43, 44), HPV (45), and focal adhesion (46) with cancer, it could be inferred that cancer pathways may play an important role between BD and AS.

By comprehensive analysis of gene expression profiles, CX3CR1 and ST6GAL1 were identified as two key hub genes between BD and AS. CX3CR1, a crucial chemokine receptor in the G protein-coupled receptor superfamily (47), is the only proinflammatory leukocyte receptor specific for the chemokine fractalkine (CX3CL1) (48), and the preservation of normal CX3CL1/CX3CR1 signaling seems to be essential for normal brain function. The expression of CX3CR1 is largely restricted to microglia within the brain parenchyma, where they play a role in remodeling neuronal circuits, serving as resident phagocytic cells involved in immune-mediated defense mechanisms, clearing damaged cell debris, and contributing to the regulation of homeostatic synaptic plasticity (49, 50). In the absence of normal CX3CL1/CX3CR1 signaling, aberrant microglial activation and elevated microglial proinflammatory activity could increase neurotoxicity, since CX3CL1/CX3CR1 signaling decreases the overproduction of inducible nitric oxide synthase, interleukin (IL)-1β, tumor necrosis factor-α (TNF-α), IL-6, and mediators of oxidative stress (48, 51). As one of the chemokines, CX3CL1 can regulate the population of central nervous system (CNS) tissue with peripherally derived cell types by relying on the activation of the CX3CR1 receptor and inhibiting the migration of astrocytes through the PI3K activity (52). Previous findings supported the notion that CX3CL1/CX3CR1 communication serves as an “off” signal, maintaining microglia in a “resting” state (53, 54).

CX3CR1 has been shown to be associated with neurodegenerative disorders and to exhibit neuroprotective effects (48). Recent conceptualizations characterize BD as a neurodegenerative disorder, marked by the progressive deterioration of brain volumes and accelerated brain aging (55). Consistent with the results of this study, significantly reduced CX3CR1 transcript level was observed in patients with BD relative to controls (56). Another study suggested that psychiatric therapy activates CX3CR1 (40), which indicated that psychiatric therapy can play a therapeutic role in reducing inflammatory response. It was speculated that the relationship between CX3CR1 and BD may be attributed to abnormal neuroinflammatory conditions. For the role of CX3CR1 in AS, several lines of evidence implicated CX3CL1/CX3CR1 in the pathogenesis of vascular inflammation injury (47). It could be observed that CX3CL1/CX3CR1 was activated by inflammatory stimuli, including TNF-a, IFN-γ, and LPS (57). CX3CL1 could act as a classical chemoattractant for T lymphocytes (58), and dendritic cells (59), which was consistent with our immune infiltration results. As chemokines and adhesion molecules, CX3CL1/CX3CR1 can directly mediate the interaction between inflammatory cells and vascular cells, and promote the plaque formation and development (47). Considering that the decrease of CX3CR1 in CNS will promote the occurrence of inflammation, the inflammatory situation may be a common physiological mechanism of BD and AS. Consistent with previous studies (60, 61), CX3CR1 had been shown to be upregulated in the AS validation group in this study. Interestingly, CX3CR1 exhibited opposite changes in BD and AS, which may be due to differences in sampling locations and the differential expression levels in the brain and vascular cells.

ST6GAL1 has anti-inflammatory effects by catalyzing sialylation of other molecules including receptors, lectins, and cytokines (62, 63). The lack of ST6GAL1 represents an excessive inflammatory response, with higher levels of neutrophilic and eosinophilic response (62, 64). As we found in this study, ST6GAL1 levels were lower than normal in BD patients, which may have contributed to the excessive of inflammation in BD patients. Large scale association analysis identified ST6GAL1 as a protective effect on CVDs occurrence (65). ST6GAL1 is strongly expressed in large blood vessels, including the aorta, and related to the angiogenic process (66). A previous study had shown that the overexpression of ST6GAL1 strongly inhibits monocyte-transendothelial migration, suggesting that ST6GAL1 could be a potential target for atherosclerosis prevention and treatment (67). However, the role of ST6GAL1 between BD and AS is not yet clear, and more research is needed to elucidate the direct relationship between them. Given that both conditions entail abnormal immune responses, the interplay between CX3CR1 and ST6GAL1 may serve as a key mechanism to understand the association between BD and AS.

It should be noted that two hub genes both related to cancer, which were identified as the key pathway through KEGG enrichment analysis in this study. CX3CL1/CX3CR1 has a tumor-suppressive activity by recruiting antitumoral immune cells such as NK and T cells into the tumor microenvironment to control tumor growth (68). Previous study observed that CX3CR1 ectopic expression improved the recruitment of adoptively transferred T cells toward CX3CL1-generated cancers, leading to the augmentation of T-cell infiltration and reduction of tumor growth (69). In addition, CX3CL1/CX3CR1 axis (70) and ST6GAL1 (71) has been confirmed to mediates several cellular functions, including activation of PI3K-Akt. ST6GAL1 has become increasingly dominant in sialyltransferase activity, which are implicated in cancer (72). ST6GAL1 is known to promote growth, survival, and metastasis, and it is upregulated in various types of cancer (including pancreatic, prostate, breast, and ovarian cancer) (73–76), while being downregulated in hepatocellular carcinoma (77). ST6GAL1 expression is thought to be associated with increased invasiveness and metastasis (78). Knockout ST6GAL1, lacking the α2,6-sialylation enzyme, is shown to exhibit impaired tumor angiogenesis through enhanced endothelial apoptosis (79). Patients with BD are more likely to develop malignant cancer than the general population, which may imply a genetic overlap in neurodevelopment and malignancy pathogenesis (80). Besides, AS are major risk factors for cancer (81). Therefore, the cancer pathway may serve as a bridge between BD and AS.

It is important to mention that, BD has been linked to clinical signs of accelerated aging, potentially explaining its association with age-related medical conditions including cancer (82). Aging is characterized by the functional decline of the immune system and is the primary risk factor for infectious diseases, CVDs, cancer, and neurodegenerative disorders (83). Given that arterial stiffness in AS is often considered a signal of vascular aging (11), it is possible that aging could be a co-pathogenesis of the two diseases. Further research on the biological mechanisms of aging and cancer between BD and AS is needed. Furthermore, both CX3CR1 and ST6GAL1 have been linked to cognitive impairment (84, 85). Among the co-regulated TFs, CREB1 has been identified as a risk factor for cognitive impairment in patients with BD (86). Given the unambiguous correlation between BD and AS and cognitive impairment (87, 88), cognition may serve as a critical connecting symptom between BD and AS.

## 5 Limitation

While we have speculated on the potential association between BD and AS based on bioinformatics analysis, there are some limitations to consider. Firstly, the absence of confounding variables in the GSE12649 dataset made it difficult to assess the stability of the differential analysis. Secondly, the DisGeNET database utilized in this study allows users to obtain genes relevant to particular diseases (89). However, this approach may filter out some potentially valuable molecules. In addition, the different sampling locations limit the interpretability of hub genes. Finally, considering feature selection algorithms appear to possess poor reproducibility in different datasets, wet experiments are needed to further verify the predictions.

## 6 Conclusions

In the present study, we utilized bioinformatic techniques including three machine learning approaches to identify 2 hub genes, CX3CR1 and ST6GAL1, which were both significantly related to BD and AS. Furthermore, we uncovered that the co-pathogenesis of the two diseases lies in the cancer-related pathways through GSEA analysis. Overall, the newly discovered diagnostic genes and potential molecular mechanisms in this study offer new clinical insights and guidance for diagnosing and treating BD and AS patients. However, further experimentation is needed to confirm the conclusions.

## Data Availability

The datasets presented in this study can be found in online repositories. The names of the repository/repositories and accession number(s) can be found in the article/**Supplementary Material**. Further inquiries can be directed to the corresponding author/s.
